# Luciferin production and luciferase transcription in the bioluminescent copepod *Metridia lucens*

**DOI:** 10.7717/peerj.5506

**Published:** 2018-09-14

**Authors:** Michael Tessler, Jean P. Gaffney, Jason M. Crawford, Eric Trautman, Nehaben A. Gujarati, Philip Alatalo, Vincent A. Pieribone, David F. Gruber

**Affiliations:** 1 Sackler Institute for Comparative Genomics, American Museum of Natural History, New York, NY, USA; 2 Department of Natural Sciences, City University of New York, Bernard M. Baruch College, New York, NY, United States of America; 3 Biology, City University of New York, Graduate School and University Center, New York, NY, United States of America; 4 Department of Chemistry, Yale University, New Haven, CT, United States of America; 5 Biology Department, Woods Hole Oceanographic Institution, Woods Hole, MA, United States of America; 6 Cellular and Molecular Physiology, Yale University, New Haven, CT, United States of America

**Keywords:** Coelenterazine, Luciferase, Coelenteramine, Luciferin, *Metridia lucens*, Copepoda

## Abstract

Bioluminescent copepods are often the most abundant marine zooplankton and play critical roles in oceanic food webs. *Metridia* copepods exhibit particularly bright bioluminescence, and the molecular basis of their light production has just recently begun to be explored. Here we add to this body of work by transcriptomically profiling *Metridia lucens*, a common species found in temperate, northern, and southern latitudes. In this previously molecularly-uncharacterized species, we find the typical luciferase paralog gene set found in *Metridia*. More surprisingly, we recover noteworthy putative luciferase sequences that had not been described from *Metridia* species, indicating that bioluminescence produced by these copepods may be more complex than previously known. This includes another copepod luciferase, as well as one from a shrimp. Furthermore, feeding experiments using mass spectrometry and ^13^C labelled L-tyrosine and L-phenylalanine firmly establish that *M. lucens* produces its own coelenterazine luciferin rather than acquiring it through diet. This coelenterazine synthesis has only been directly confirmed in one other copepod species.

## Introduction

Copepods are small crustaceans (generally ∼1–2 mm) that are a primary component of marine zooplankton communities, holding key roles in oceanic food webs (Takenaka, Yamaguchi & Shigeri, 2017). These animals act as lower-order consumers, important grazers on microplankton, and as a major food source for many invertebrate and vertebrate predators, such as cephalopods and fishes (Marine Zooplankton Colloquium 2, 2001; Takenaka, Yamaguchi & Shigeri, 2017). Globally, the bulk of plankton communities (generally 55–95% of the individuals) are often populated by copepods (Longhurst, 1985). Within the zooplanktonic copepods, the order Calanoida can dominate (e.g., in the Arctic Ocean) in terms of biomass (Kosobokova & Hirche, 2009; Forest et al., 2011). Given the exceptional biomass and low-level trophic positioning of the calanoids, these tiny drifters themselves act as an important component of aquatic foodwebs (Takenaka, Yamaguchi & Shigeri, 2017).

**Figure 1:**
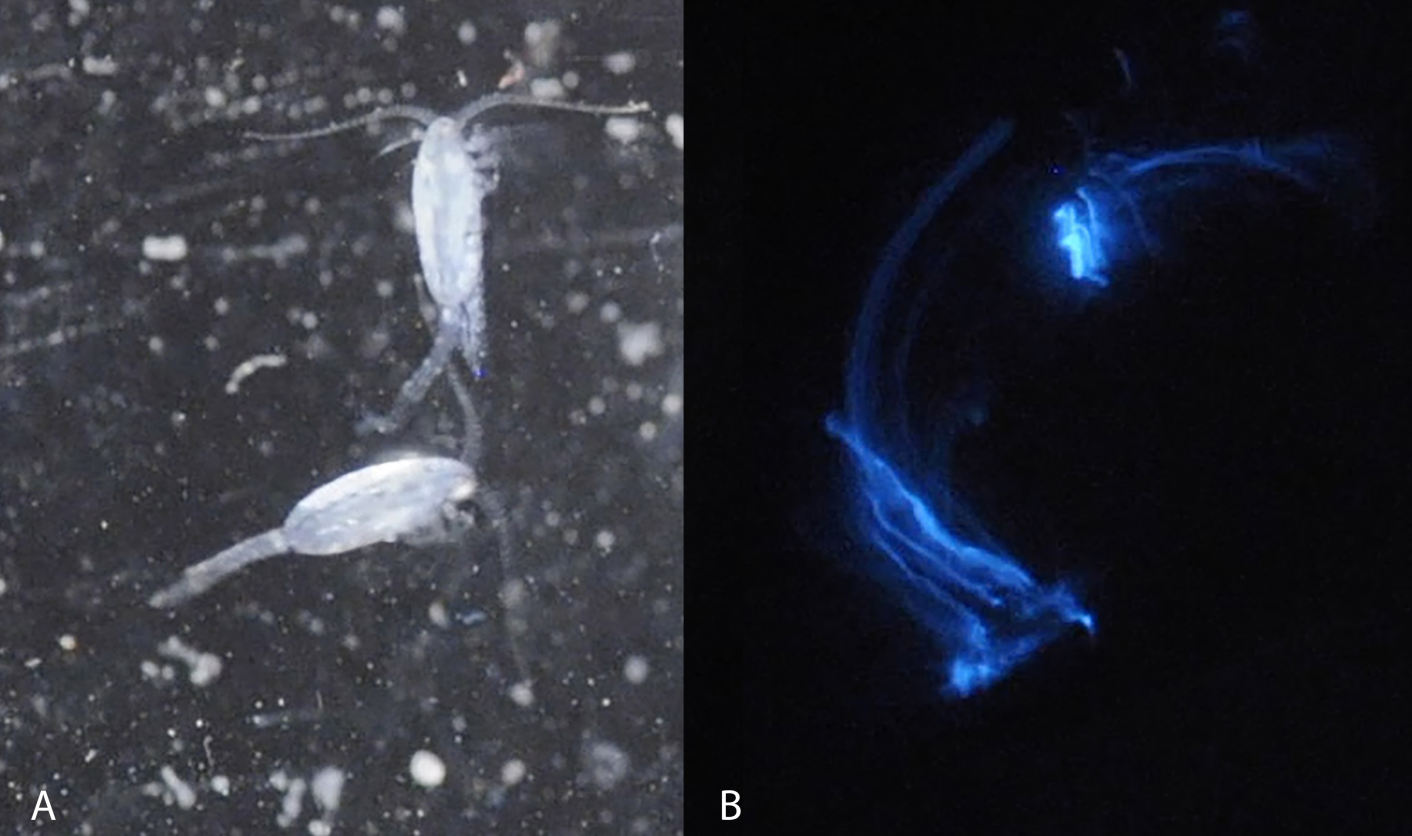
Light microscopy of *Metridia lucens.* Under normal light (A) and while excreting bioluminesce (B).

Within the calanoid copepods, bioluminescent species can comprise much of the life (5–59% of the abundance and 10–15% of the biomass) in marine ecosystems (Takenaka, Yamaguchi & Shigeri, 2017). Species in the superfamily Augaptiloidea are known for their bioluminescence, and in at least one comparison they produced the highest bioluminescent activity of any calanoids (Takenaka et al., 2012). Species in the genus *Metridia* have exceptionally bright bioluminescence (Takenaka et al., 2012). Several *Metridia* species have been reviewed regarding the molecular basis of their bioluminescent activity, finding interesting patterns of luciferase gene duplication within the Metridinidae (Takenaka et al., 2012). Still, most of the species remain poorly studied or are uncharacterized in this regard.

Blue bioluminescence produced by *Metridia lucens* (Fig. 1 and Supplemental Information) was first noted when the species was described over 150 years ago (Boeck, 1864), yet its bioluminescence still remains uncharacterized molecularly. This moderately sized copepod (2.4–3.0 mm for females and 1.8–2.5 mm for males) is common in temperate to more northerly waters, as well as southerly waters (David & Conover, 1961; Stupnikova et al., 2013). Both anterior and posterior regions of *M. lucens* bioluminesce, ultimately producing a bright flash that tapers off over a few seconds but can be activated repeatedly (David & Conover, 1961). The bioluminescent flashes produced by *M. lucens* can be triggered by disturbance to the water and possibly by the presence of predators (David & Conover, 1961).

The two primary photoreceptors found in Crustacea are naupliar eyes and compound eyes. *Metridia lucens* and all copepods use naupliar eyes which are traditionally considered simpler structures that function in phototaxis, rather than in image formation (Ali, 2013). However, naupliar eyes are not always simple and can be highly specialized in some groups of copepods (Porter et al., 2017). Despite not having image-forming eyes, copepods react strongly when exposed to simulated bioluminescence (Buskey & Swift, 1985; Widder & Johnsen, 2000). Like several other copepods, *M. lucens* increase their swimming speed and spiral off photophobically in an overall straight path when exposed to such flashes (Buskey & Swift, 1985).

In the Gulf of Maine, *M. lucens* densely aggregate, forming bioluminescent “hot spots” that are vertically constrained to a half meter (Widder et al., 1999). Given the response of *M. lucens* to physical disturbance, the half meter band where they aggregate acts as a “bioluminescent minefield” (Widder & Johnsen, 2000; Widder, 2002). It has been hypothesized that when a larger animal disturbs the *M. lucens* aggregation, it will trigger the copepods to illuminate the water and expose this larger animal to even larger predators that may be lurking (Widder & Johnsen, 2000; Widder, 2002). While the exact mechanism should be tested further, it is evermore apparent that bioluminescence in *M. lucens* is at least in part some form of predator evasion.

In this study we seek to characterize molecular aspects of bioluminescence in the copepod *M. lucens*. To accomplish this, we performed Illumina-based transcriptome sequencing of this species to find RNA sequences of luciferase enzymes and to search for putative luciferin producing sequences. Furthermore, we review this species for biomolecular products relating to the luciferins used as substrate for bioluminescence.

## Materials and Methods

### Sample collection

*Metridia lucens* samples were collected on January 12, 2016 using the *R/V Tioga* (Woods Hole Oceanographic Institution) on Cape Cod Bay in waters of 30–40 m depth. Their identities were confirmed morphologically and with COI and 18S transcriptomic sequences. A $ \frac{3}{4} $-m plankton net with 243 µm mesh netting was deployed and a double-oblique tow was conducted to 2 m off the bottom. Contents of each tow were diluted into ambient seawater in 30-liter barrels or into insulated foam coolers for transport to the laboratory. Within four hours, plankton was transferred to a walk-in cold-room for sorting (at a rate of 50 copepods/hour). Cultured phytoplankton (a mixture of diatoms, flagellates, and dinoflagellates) were fed to the copepod cultures, held in 12-L glass carboys, with diffuse air-stones for aeration at 8 °C, and a photoperiod of 13L/11D. Cultures were changed every three days and checked for spawned eggs. Eggs were removed and cultured separately, as adults are known to be cannibalistic on eggs and nauplii.

### Transcriptomic search for bioluminescence related sequences

RNA was extracted from 10 individuals of *M. lucens* using an RNeasy Fibrous Tissue Mini Kit (Qiagen #74704); males and females were mixed in the collection used. Extracted RNA was then used for transcriptome sequencing at the New York University Langone Medical Center Genomics Technology Center using an Illumina HiSeq 2500 with paired-end 100 bp sequences (using V4 chemistry). The resulting sequences were processed using our prior protocols (Tessler et al., 2018), which are briefly summarized here. First, sequences were trimmed for quality control using Trimmomatic (Bolger, Lohse & Usadel, 2014) and then assembled using Trinity 2.4 (Grabherr et al., 2011; Haas et al., 2013) with default parameters. Open reading frames (ORFs) were then predicted from the assembled transcriptome using Transdecoder 3.0 (Haas et al., 2013), with a minimum requirement of 5 amino acids due to our interest in potentially short luciferins.

ORFs were then queried against three local databases. The first and primary local database consisted of crustacean luciferase sequences from GenBank, with a focus on copepod luciferases. The second local database consisted of photoprotein sequences used in Brugler et al. (2018). The third local database consisted of potential luciferins and closely related isopenicillin-N-synthase sequences. The putative luciferin sequences in this third database ended in the amino acids “FYY”, were found to be similar to isopenicillin-N-synthase, and were identified as putative luciferin biosynthesis proteins in prior work (Francis et al., 2015). For each query against these local databases we used blastp with e^−5^ set as an *e*-value minimum; only the top matching hit was retained for further review. To insure that no other proteins were better matches to ORFs putatively matching a sequence in our local database, we used these ORFs as queries against the Swissprot/Uniprot database using blastp. The Swissprot/Uniprot database was used here rather than other databases because it is well-curated. When bitscores from the local database queries were equal or higher than the results from Swissprot/Uniprot, the sequence were considered a putative match to the protein of interest (e.g., candidate luciferases). Bitscores were used, as they avoid issues of differing database size (here our local database vs. Swissprot/Uniprot) inherent in *e*-value cutoffs.

### Phylogenetics of *Metridia*-related Luciferases and COI

In order to produce a *Metridia*-related luciferase gene tree, luciferase sequences utilized for a prior phylogenetic reconstruction (Takenaka et al., 2012) were combined with our ORF sequences that best matched *Metridia* sequences from that study; specifically, the single longest ORF per Trinity read cluster. Alignments of these sequences were then produced using MUSCLE v3.8.31 (Edgar, 2004). A model of amino acid replacement was selected (WAG + G) for the resulting alignment using ProtTest 3.4.2 (Darriba et al., 2011). A phylogenetic reconstruction with 1,000 bootstrap replicates for support was then produced using the aligned amino acid sequences and selected model in RAxML 8.2.10 (Stamatakis, 2014) in the CIPRES Science Gateway (Edgar, 2004; Miller, Pfeiffer & Schwartz, 2010). Outgroup taxa (*Heterorhabdus tanneri*, *Heterostylites major*, and *Lucicutia ovaliformis*) for the resulting luciferase gene tree were selected based on a previous luciferase phylogenetic reconstruction of bioluminescent copepods (Takenaka et al., 2012).

As the taxonomic status of *M. lucens* has been debated in the literature, we generated a phylogenetic matrix of the COI barcoding locus for *Metridia* (Supplemental Information). Sequences downloaded from GenBank had their primers trimmed when necessary. Phylogenetic reconstruction follows that of the luciferases, except nucleotides were used and jModelTest 2.1.4 (Darriba et al., 2012) determined a model (GTR + I +Γ). The outgroups were furthermore based on the 18S phylogeny of bioluminescent copepods (Takenaka et al., 2012).

### Isotope experiment

^13^C labelled L-tyrosine and L-phenylalanine (Cambridge Isotopes CLM-2263 and CLM-2250) were added to seawater in a final concentration of 10 mg/L together with live *M. lucens* specimens. The copepods were not fed once they were transferred to seawater containing isotopes. Copepods remained in the labelled seawater for 4 days. On Day 4, nine individuals were collected from the control, five from the phenylalanine culture, and one from the tyrosine culture. The whole organisms were frozen immediately upon collection*. Metridia lucens* were lyophilized as received. The remaining solids were extracted with MeOH +1 M HCl. Extracts were analyzed by LC-MS using an Agilent iFunnel 6550 Quadrupole-Time-of-Flight Mass Spectrometer (Q-TOF).

## Results

### Transcriptomic search for bioluminescence related sequences

Raw transcriptomic reads are deposited in the Short Read Archive under accession SRX3899629. The assembled transcriptomic sequences had 308,066 contigs, that resulted in 237,343 ORFs. Of the luciferase sequences in our local database, 10 had matching ORFs that were retained after reciprocal BLAST searches against Swissprot/Uniprot (Table 1 and Supplemental Information). Of these, nine were matches to other species in the copepod; specifically, *Heterorhabdus tanneri*, *Metridia okhotensis*, *Metridia pacifica*, and *Pleuromamma* sp. Another sequence matched a bioluminescent shrimp (*Oplophorus gracilirostris*).

**Table 1:** Crustacean luciferases that match sequences from the transcriptome of *Metridia lucens*.

**Taxon**	**GenBank accession**	**Percent identity**	**Matching length**	*E*-value	**Bitscore**
*Heterorhabdus tanneri*	BAL63040	36.8	38	5.4E−06	33.5
*Metridia okhotensis*	BAL63033	97.2	144	2.3E−106	293
*Metridia pacifica*	BAD93334	96.9	163	10.0E−117	320
*Metridia pacifica*	BAY00650	100	96	1.4E−69	198
*Metridia pacifica*	BAY00651	100	92	2.0E−66	190
*Metridia pacifica*	BAG48249	78.5	65	3.6E−31	99.4
*Metridia pacifica*	BAD93333	97.6	41	1.5E−25	84
*Metridia pacifica*	BAY00656	94.7	19	4.4E−11	44.7
*Oplophorus gracilirostris*	Q9GV46	25.5	376	1.3E−23	92
*Pleuromamma* sp.	AAG54096	62.1	124	6.6E−57	167

None of the sequences matching putative luciferins were retained after reciprocal BLAST searches against Swissprot/Uniprot and none ended in “FYY” amino acid sequences, as has been suggested to be of importance (Oba et al., 2009; Francis et al., 2015). Instead these sequences generally better matched other oxidoreductases. Similarly, no photoproteins were uncovered; these better matched calmodulin or, less frequently, troponin.

### Phylogenetics of *Metridia*-related Luciferases and COI

**Figure 2:**
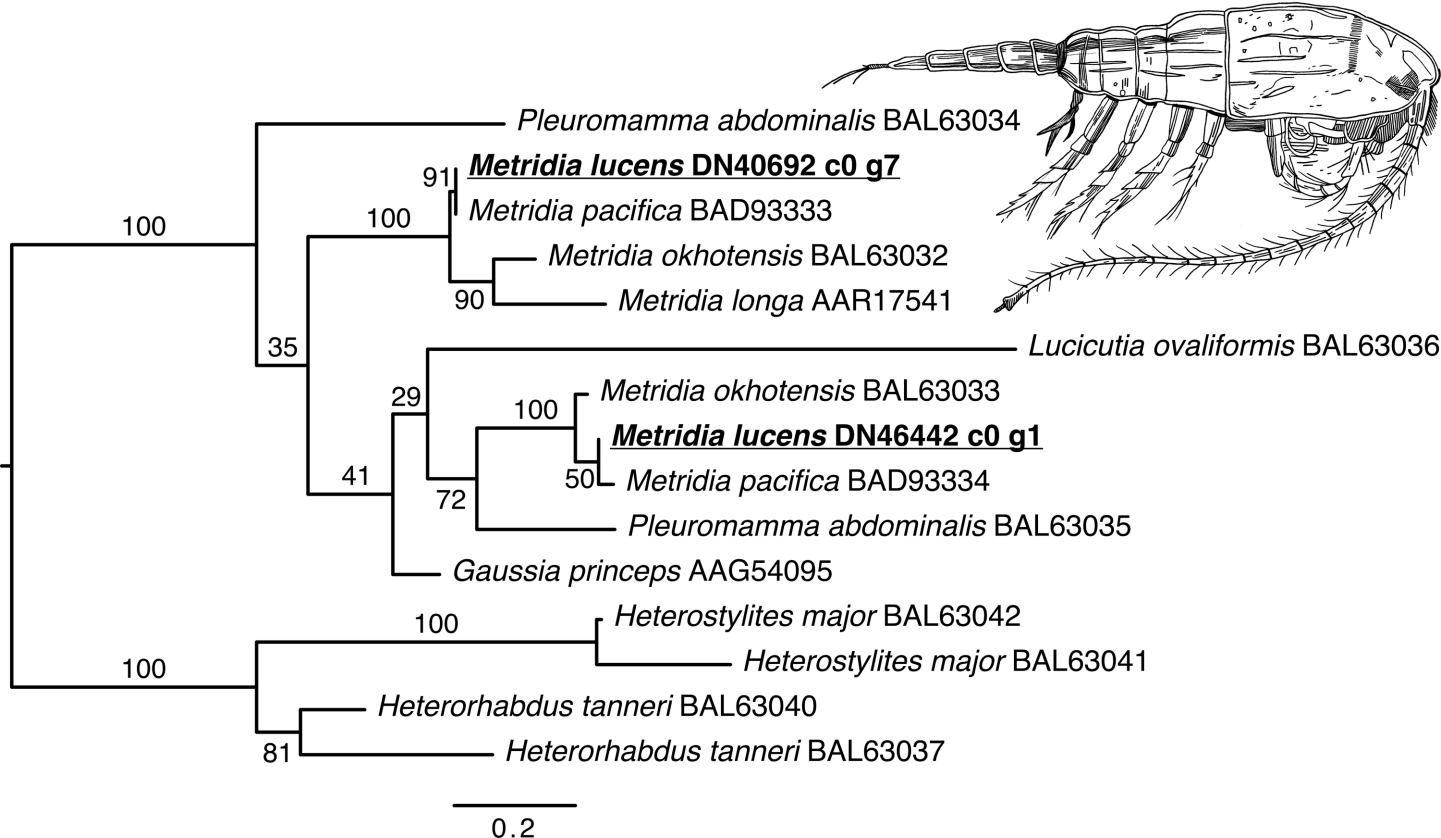
Maximum likelihood phylogenetic reconstruction of luciferases from *Metridia* species and related copepods. Support values are summaries of 1,000 bootstrap replicates. Image credit for copepod drawing: Olivia A. Gresham.

The phylogenetic reconstruction of the *Metridia*-related luciferases resulted in two principal ingroup clades, each with a paralogous pair of sequence from our ORFs (*M. lucens*), *M. okhotensis*, and *M. pacifica* (Fig. 2). One of these clades also contained single sequences for *M. longa* and *Gaussia princeps*, as well as one of the *Pleuromamma abdominalis* sequences. The *Metridia* sequences in each of these clades were well-supported as being monophyletic (bootstraps = 100%). Many other parts of the tree were poorly supported.

**Figure 3:**
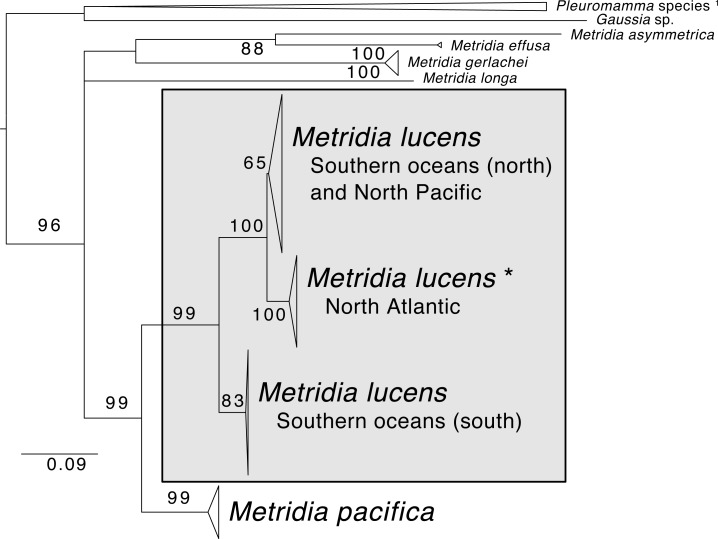
Maximum likelihood phylogeny of COI of *Metridia* copepods. Support values are summaries of 1,000 bootstrap replicates. * The North Atlantic *Metridia lucens* clade contains the COI sequence from our *M. lucens* transcriptome. ^1^In addition to the *Pleuromamma* species, a sequence of *Metridia venusta* was also found in this clade.

The phylogenetic reconstruction of the COI locus for *Metridia* species (Fig. 3 and Supplemental Information) showed clear differentiation between described species and high genetic variation within *M. lucens* (<10%). We would also like to note that a sequence labeled as *Metridia gerlachei* (HM045328) is clearly a misidentified *M. lucens* individual, and that the sequence for *Metridia venusta* nested within the *Pleuromamma* clade (either this species is misidentified or the taxonomy is in need of updating).

### Isotope experiment

**Figure 4:**
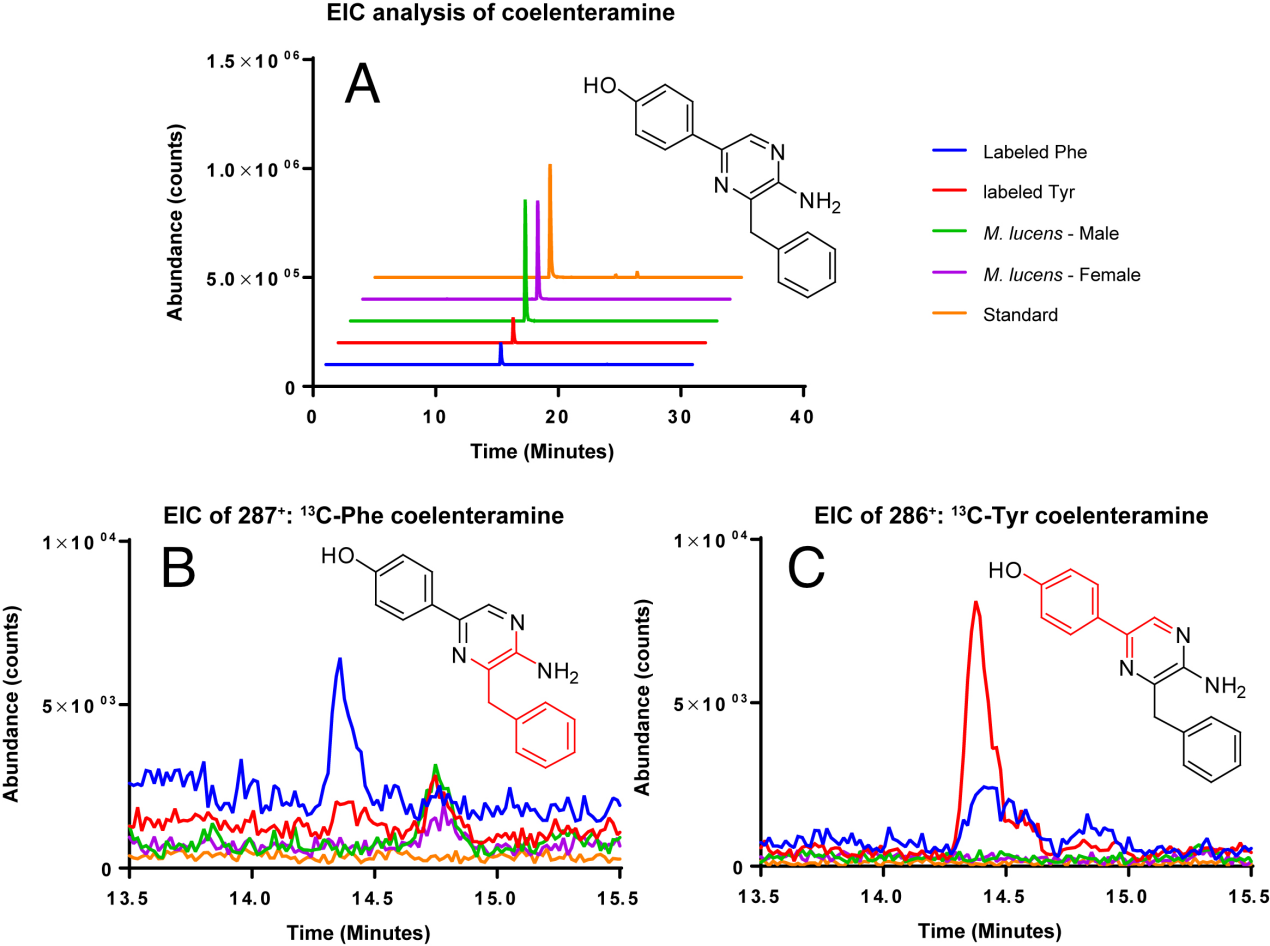
EIC analysis of the labeled coelenteramines. (A) Extracted ion count chromatograms (EICs, extraction window ±10 ppm) for natural ^12^C-coelenteramine (M + H) under ^13^C-labeling and non-labeling conditions. (B/C) EICs for the ^13^C_9_-labeled (B) and the ^13^C_8_-labeled (C) coelenteramine ions centered at *m/z* 287.1590 and *m/z* 286.1556 are shown. Data support that the carbons depicted in red are isotopically labeled at low abundance. The peak at 14.4 min has the same retention time as the coelenteramine standard. Observed for ^13^C_8_-coelenteramine: 286.1561, 1.7 ppm error. Observed for ^13^C_9_-coelenteramine: 287.1598, 2.8 ppm error.

Coelenterazine was not detected in any of the samples, likely due to the age of the copepods. However, the degradation product of coelenterazine, coelenteramine was detected (Fig. 4). Phenylalanine and tyrosine labels were observed in coelenteramine from the copepods, supporting that the copepods synthesize coelenterazine.

## Discussion

Like other bioluminescent *Metridia* species (Takenaka et al., 2012), *M. lucens* appears to transcribe luciferase enzymes that oxidize luciferins in order to produce light, and it does not appear to rely on photoproteins for this task. More surprisingly, *M. lucens* transcribes putative luciferases that have not been noted for other *Metridia* copepods. Furthermore, *M. lucens* is one of the few organisms now documented to produce its own coelenterazine luciferin, extending findings on the closely related species *M. pacifica* (Oba et al., 2009). Below we detail these findings, resulting in the first molecular characterization of bioluminescence in *M. lucens*.

### Luciferases

In our transcriptomic data, several sequences matched luciferases from a variety of crustaceans. Most interestingly, we find a luciferase from the deep-sea shrimp *Oplophorus gracilirostris*. This is notable, as no such sequence has previously been recorded from any species of *Metridia* or other copepod as far as we are aware.

We also recovered a number of sequences matching the more well-reviewed Metridinidae luciferases, corroborating prior work (Markova et al., 2004; Takenaka et al., 2008; Takenaka et al., 2012). A Metridinidae luciferase was first cloned from *Gaussia princeps* (Verhaegent & Christopoulos, 2002). Luciferases in this family have more recently been found and compared for several *Metridia* species (Takenaka et al., 2012; Takenaka et al., 2013), and have been shown to usually consist of two paralogous pairs of sequences per species. Indeed our *Metridia*-related luciferases fall out into these two clades.

Aside from the typical *Metridia* luciferases, we recovered a match from *M. lucens* to a *Pleuromamma* (Metridinidae) luciferase that had not been uncovered before from a *Metridia* species. While *Pleuromamma abdominalis* does also have the typical luciferase studied in *Metridia* (Takenaka et al., 2012), this second luciferase comes from an unidentified member of the genus and has documented bioluminescent activity (Bryan & Szent-Gyorgyi, 2001). Our finding of this luciferase in *M. lucens* expands the number of putative luciferases transcribed by the genus *Metridia*. To assess functional similarity, our sequence was modeled using Phyre2 (Kelley et al., 2015) and SWISS-MODEL (Waterhouse et al., 2018) and compared to the model generated for this *Pleuromamma* luciferase. The Pyre2 models used the same template and showed a confidence level of approximately 33% indicating some similarity, but notable difference. The SWISS-MODEL used different templates. It is worth noting that *Pleuromamma* is one of the genera most closely related to *Metridia* (Takenaka et al., 2012); however, this second luciferase from *Pleuromamma* had not been previously searched for in *Metridia* as far as we are aware.

### Luciferins

The majority of bioluminescent organisms react with the luciferin coelenterazine to produce light, including a variety of phyla and close to 100 genera (Thomson, Herring & Campbell, 1997; Haddock, Moline & Case, 2010; Markova & Vysotski, 2015). While many animals acquire the luciferin coelenterazine through diet (Haddock, Rivers & Robison, 2001), our mass spectrometry confirms that *Metridia* species appear to be some of the few known animals to produce coelenterazine (Buskey & Stearns, 1991; Oba et al., 2009). In prior work it was established that *M. pacifica* uses an L-phenylalanine and two L-tyrosine molecules to produce said coelenterazine, possibly with: “autocyclization of tri-peptides such as H_2_N-Phe-Tyr-Tyr-COOH” (Oba et al., 2009). This study confirms that this second species of *Metridia*, *M. lucens* (or a symbiont it houses), also synthesizes coelenterazine *de novo*.

The promise of coelenterazine being a cyclized “Phe-Tyr-Tyr” (“FYY”) led researchers to search for these amino acids at the end of peptides in ctenophores, another animal group believed to produce their own coelenterazine (Francis et al., 2015). This resulted in some exciting findings, with C-terminus “FYY” proteins that best matched oxidoreductases: isopenicillin-N-synthases or oxygenases. Furthermore, these “FYY” proteins were found across all bioluminescent species, but were absent from non-bioluminescent ones. Still, while these ctenophore putative oxidoreductases are promising, they have not been expressed or knocked out to confirm function.

None of our ORFs better matched the C-terminus “FYY” oxidoreductases as compared to Swissprot/Uniprot. Furthermore, while we recovered three (Supplemental Information) mature transcripts (here considered to have a start and stop codon) with C-terminus “FYY” sequences, these did not produce any clear matches in BLAST searches (i.e., no well-described matches had an *e*-value below our cutoff). Furthermore, to compare structure, we modeled these ORFs with SWISS-MODEL (Waterhouse et al., 2018). This generated dissimilar models, further supporting that these transcript do not share structural similarities with other proteins. However, it is only the most plausible hypothesis thus far put forward that C-terminus “FYY” is required to produce coelenterazine; these amino acids could be produced by other routes. Regardless, there is currently no clear molecular path indicating how *M. lucens* might produce coelenterazine, just that it does produce it.

### A taxonomic note

Whether *M. lucens* and *M. pacifica* are one widely distributed species or are indeed two distinct species has been debated in the taxonomic literature (Bucklin, Frost & Kocher, 1995). Yet, based on recent molecular studies, the two species appear to be genetically separated by 12.6–14.4% for the COI barcoding locus (Blanco-Bercial et al., 2014). Furthermore, there is notable (∼8%) genetic differentiation even for lineages within *M. lucens*, suggesting that this taxon alone is composed of several species (Stupnikova et al., 2013; Blanco-Bercial et al., 2014). To contextualize this, studies generally consider crustaceans with ∼3% genetic divergence in COI to indicate that lineages are separate species (Stupnikova et al., 2013). Adding to this evidence, we combined COI sequences of *Metridia* from GenBank and found highly similar results, with *M. lucens* having several distinct clades within it and being genetically distant from *M. pacifica* (10.1–14.6% for COI distance). Our *M. lucens* sample clearly nest within the North Atlantic clade. Future studies should strive to review the lineages within *M. lucens* and determine if morphological traits can be found for describing them as new species.

## Conclusions

Based on our findings, the molecular mechanisms of *M. lucens* bioluminescence appear to be more complex than reported (Takenaka et al., 2012) for other species in the genus *Metridia*. Most likely this is not unique to *M. lucens*, but instead *Metridia* and even other genera in the family Metridinidae may have the additional putative luciferases that we recovered for *M. lucens*. Future work should explore the interaction of these bioluminescent loci across Metridiniadae.

##  Supplemental Information

10.7717/peerj.5506/supp-1 Supplemental Information 1 Video of bioluminescent *Metridia lucens* Specimens first under normal light and then while excreting bioluminesce.

10.7717/peerj.5506/supp-2 Supplemental Information 2 Fasta file of COI alignment for *Metridia* species Samples are identifiable by GenBank accession numbers.

10.7717/peerj.5506/supp-3 Supplemental Information 3 Fasta file with putative luciferase sequences

10.7717/peerj.5506/supp-4 Supplemental Information 4 Tree file of COI phylogeny for *Metridia* species Samples are identifiable by GenBank accession numbers.

10.7717/peerj.5506/supp-5 Supplemental Information 5 Fasta file with mature C-terminus “FYY” sequences

## Additional Information and Declarations

### Data Availability

The following information was supplied regarding data availability: Raw reads from the *Metridia lucens* transcriptome are deposited in the Short Read Archive under accession SRX3899629 (BioProject PRJNA449123).
